# Preliminary Investigation on the Effects of Squeeze-Hold Intervention on Muscle Soreness Relief

**DOI:** 10.7759/cureus.91212

**Published:** 2025-08-28

**Authors:** Masaaki Nakajima

**Affiliations:** 1 Physical Therapy, School of Health Science and Social Welfare, Kibi International University, Takahashi, JPN

**Keywords:** delayed-onset muscle soreness (doms), microcirculation, muscle pain relief, pneumatic cuff, squeeze-hold therapy

## Abstract

Background: Delayed-onset muscle soreness (DOMS) occurs 24-48 hours after exercise and is associated with muscle fiber damage and inflammation. Although various treatments have been proposed, only a few have demonstrated consistent efficacy. This pilot study investigated the immediate effects of squeeze-hold therapy on DOMS using a pneumatic cuff.

Methods: Ten healthy collegiate students (mean age: 20.8±1.4 years; body mass index: 22.8±2.2 kg/m^2^) were recruited. DOMS was induced in the triceps surae using a heel-raise exercise. After 24 hours, a pneumatic cuff was applied to the left lower leg, inflated to 200 mmHg, and maintained for three minutes. Pain was assessed using the Talag scale immediately before, 10 minutes after, and 24 hours after the intervention (48 hours after the DOMS-inducing exercise task). The untreated right leg served as the control. Data were analyzed using Wilcoxon's signed-rank test (α=0.05).

Results: Muscle soreness was significantly reduced immediately after the intervention on the treated side (p=0.018). However, this analgesic effect was not sustained, and no significant difference was observed 24 hours later.

Conclusion: Squeeze-hold intervention produced a transient analgesic effect when applied 24 hours after DOMS-inducing exercise, likely due to reactive hyperemia and gate control mechanisms. However, soreness recurred as inflammatory mediators continued to be produced. Repeated application during the 24-48-hour period, when CRP remains elevated, may provide more consistent pain relief.

## Introduction

Squeezing the muscle with a pneumatic cuff and holding it for a certain period can promote muscle blood flow upon release [1,2]. This effect may be explained by two primary mechanisms. The first mechanism involves the release of nitric oxide (NO) due to shear stress on the vascular endothelial cells during reperfusion. Shear stress from blood flow causes NO to be released from the vascular endothelial cells. NO relaxes vascular smooth muscles and promotes blood flow [3-6]. The second mechanism involves an increase in carbon dioxide (CO_2_) concentration due to muscle cell metabolism during blood flow restriction. Cuff compression induces ischemia. By maintaining this state, the CO_2_ concentration in the muscle tissue increases because of ongoing cellular metabolism. The decrease in muscle volume (reduction in cell volume and increase in cell density) caused by squeezing further accelerates CO_2_ accumulation. The resulting decrease in pH due to elevated CO_2_ levels relaxes the vascular smooth muscle [7], thereby promoting blood flow after release.

The regulation of blood flow in muscle tissue involves arteriolar smooth muscles, which normally maintain a dormant capillary network [7,8]. Therapies such as heat or massage typically increase blood flow in active capillaries but not in dormant capillaries. In contrast, squeeze-hold therapy relaxes all precapillary sphincters in the treated area, enabling perfusion of the dormant capillary network. This leads to a superior flushing effect through the removal of metabolites and inflammatory substances.

This study aimed to investigate the effect of squeeze-hold therapy using pneumatic cuffs on the relief of delayed-onset muscle soreness (DOMS) in healthy participants [9-11].

## Materials and methods

Participants

Ten healthy college students from Kibi International University participated in this study. The average age of the participants was 20.8±1.4 years, and the average body mass index was 22.8±2.2 kg/m^2^. The study was approved by the Ethics Review Committee of Kibi International University (approval number: 21-44). Prior to the commencement of the experiment, participants received detailed explanations of the study both in writing and verbally to ensure full comprehension. Written informed consent was then obtained from all participants. Following consent, each participant completed a medical history questionnaire. Participants with any history of lower limb musculoskeletal injuries or disorders that could affect muscle soreness or recovery were excluded. 

Experimental protocol

**Figure 1:**
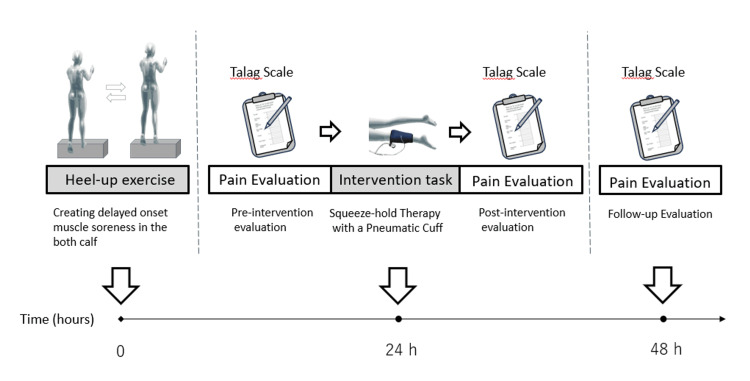
Experiment protocol The participant performs a heel-up task to generate delayed-onset muscle soreness in both lower legs. Twenty-four hours later, a pneumatic cuff squeeze-hold is applied to the left lower leg under prescribed conditions as an intervention task. Muscle soreness is evaluated using the Talag scale immediately before, 10 minutes after, and 24 hours after the intervention task.

DOMS was induced in the triceps surae muscle using a heel-raise exercise. DOMS peaks 24-48 hours after exercise [9,11]. The intervention (pneumatic cuff application) was performed 24 hours after the DOMS-inducing exercise. Pain was evaluated immediately before, 10 minutes after, and 24 hours after the intervention (Figure 1).

DOMS induction

**Figure 2:**
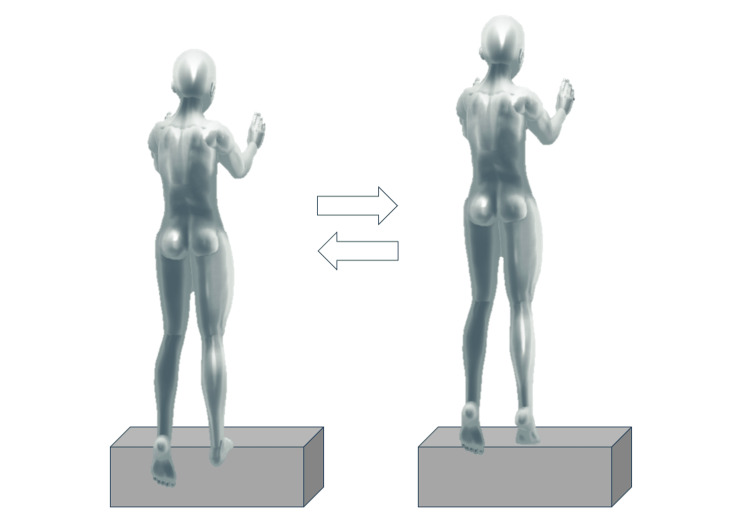
Heel-up exercises The participant climbs onto a standing platform and balances himself by placing his hands on the wall in front of him. One foot is held out behind the step platform. The toe of the other foot is placed over the platform, and the heel is out from the platform. From this state, the ankle joint is plantar-dorsiflexed to the sound of a metronome, and a heel-up exercise is performed. Heel-up exercises are performed on both lower extremities, one leg at a time, under the condition of 10 sets of 30 at a speed of 0.5 Hz. A one-minute break is observed between sets.

Based on a previously described protocol [12], participants performed 10 sets of 30 repetitions of heel-raises at a frequency of 0.5 Hz, with a one-minute rest between sets. The exercises were performed separately for each leg. The participants were instructed to exert maximal effort. An electric goniometer attached to the ankle joint was used to monitor the range of motion, and the data were recorded using PowerLab (ADInstruments, Dunedin, New Zealand). If a participant failed to achieve at least 50% of the maximum plantar dorsiflexion range for five consecutive repetitions, the set was considered complete. This criterion was also used to determine the end of the subsequent sets (Figure 2).

Intervention

**Figure 3:**
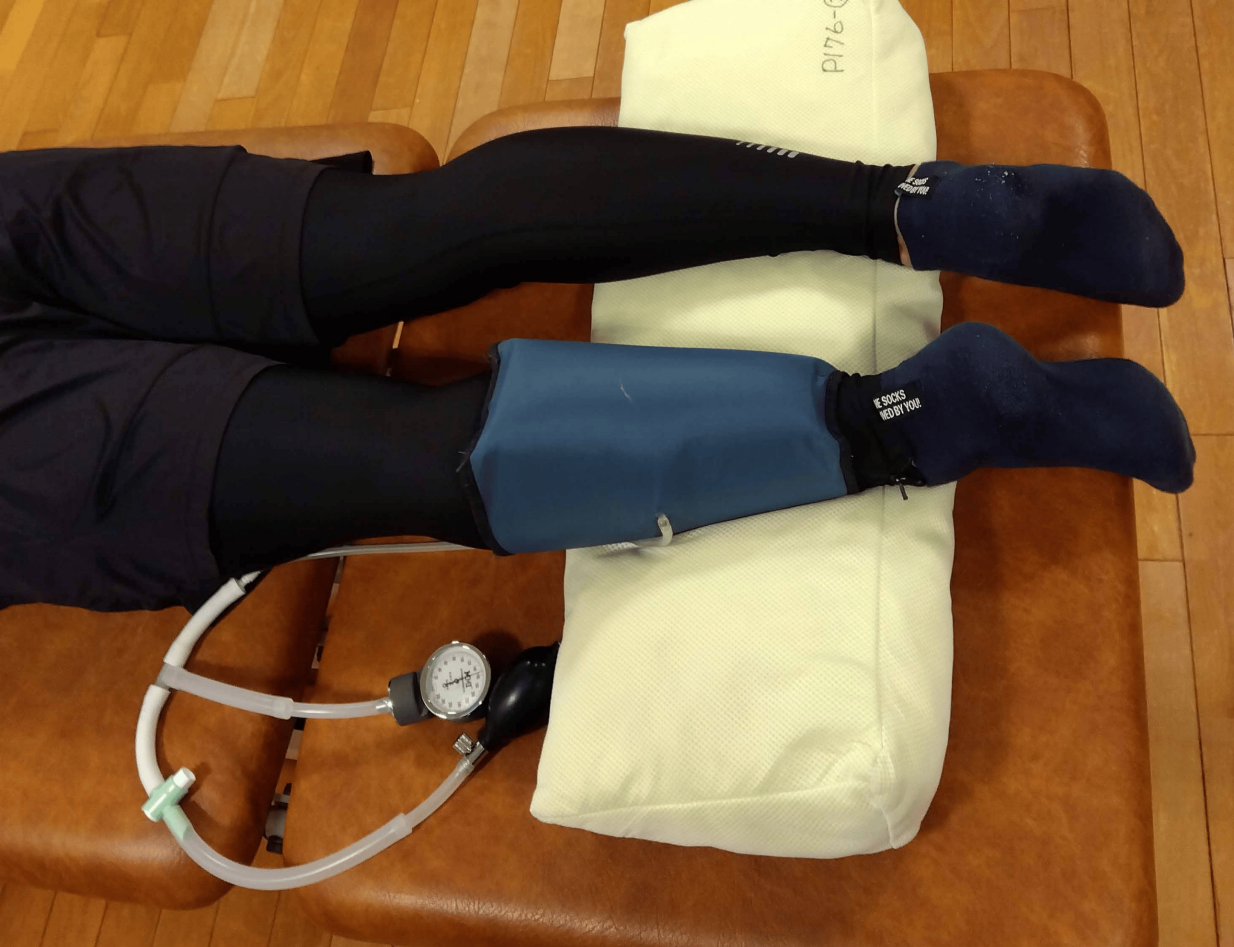
Pneumatic cuff The pneumatic cuff is made of sturdy nylon and is sized to cover the lower leg. An internal air chamber is present in it, which applies pressure on the gastrocnemius muscle. The size of the air chamber is 13 cm on the top, 9 cm on the bottom, and 22 cm on both vertical sides. Velcro makes it easy to put on and take off. The valve on the surface of the cuff enables the insertion of air. A pneumatic cuff is wrapped around the participant's left lower leg as they assumed a prone position.

Twenty-four hours after DOMS induction, a pneumatic cuff was applied to the left lower leg, pressurized to 200 mmHg, and maintained for three minutes (Figure 3). Subsequently, the cuff was deflated, and the participant rested for 10 minutes. This protocol has been previously shown to enhance muscle blood flow for at least 10 minutes after release [2].

Pain assessment

**Figure 4:**
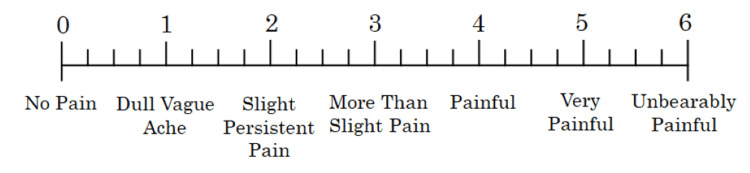
Talag scale The Talag scale measures delayed-onset muscle soreness at 25 levels using a 7-point main scale and 4-point subscale. The higher the number, the greater is the degree of muscle pain. This figure is adapted with modifications from [14].

Pain in both lower legs was evaluated immediately before and 10 minutes after the intervention, and 24 hours after the intervention, using the Talag scale, a validated tool for assessing DOMS-related pain (Figure 4) [13,14].

Data analysis

Pain scores in the intervention side and the non-intervention side were compared using Wilcoxon's signed-rank test. Statistical significance was set at p<0.05. 

## Results

**Figure 5:**
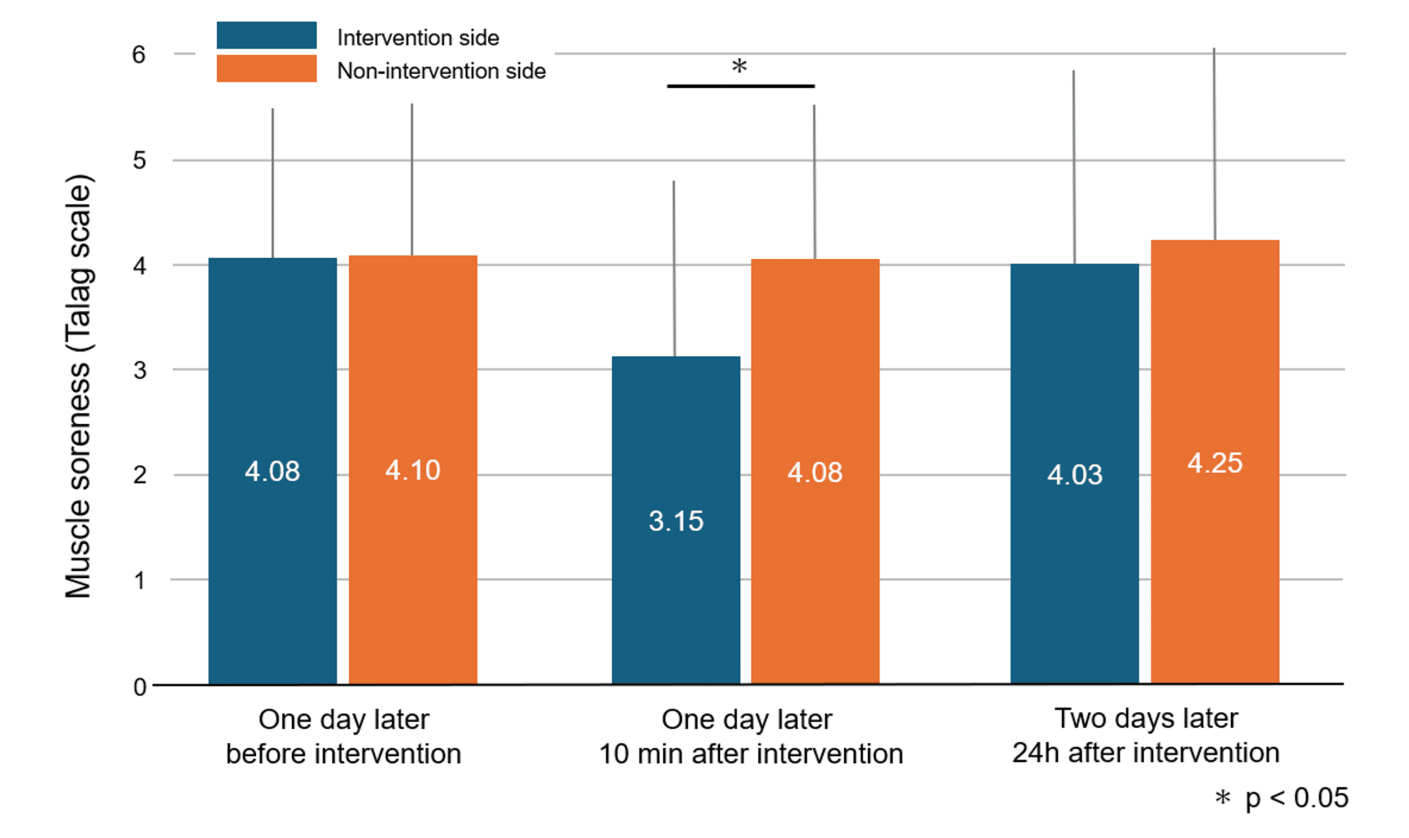
Muscle soreness There was no significant difference in muscle soreness before the intervention between the intervention side and the non-intervention side. Ten minutes after the intervention, muscle soreness was significantly lower in the intervention side (*p=0.018). Twenty-four hours after the intervention, there was no significant difference in muscle soreness between the intervention side and the non-intervention side.

All participants completed the prescribed DOMS-inducing exercise and the subsequent intervention protocol without adverse events. The mean Talag scale score on the intervention side was 4.08±1.41, 3.15±1.66, and 4.03±1.83, respectively, immediately before, 10 minutes after, and 24 hours after the intervention. Similarly, the mean Talag scale score on the non-intervention side was 4.10±1.43, 4.08±1.45, and 4.25±1.81, respectively. Ten minutes after the intervention, the intervention side showed a significantly lower pain score compared to the non-intervention side (p=0.018) (Figure 5).

## Discussion

In this study, the squeeze-hold intervention was performed 24 hours after DOMS-inducing exercise, and pain scores were significantly lower on the intervention side than on the non-intervention side, indicating that squeeze-hold therapy alleviated DOMS. However, 24 hours after the intervention, there was no longer a significant difference in pain scores between the intervention side and the non-intervention side, indicating that squeeze-hold therapy is no longer effective.

DOMS is characterized by muscle pain that develops 24-48 hours after unaccustomed or intense exercise, especially those involving eccentric contractions. This is thought to result from muscle fiber damage and subsequent inflammation [11,15]. Inflammatory mediators such as prostaglandins lower the pain threshold, leading to soreness. Although many treatment methods for DOMS have been explored, few have been proven consistently effective [11]. The present study demonstrated that the squeeze-hold intervention performed 24 hours after DOMS-inducing exercise transiently reduced muscle soreness; however, the analgesic effect diminished after another 24 hours. This transient effect may be explained by two mechanisms. First, the squeeze-hold maneuver elicited reactive hyperemia, leading to an acute increase in muscle blood flow and facilitating the clearance of inflammatory mediators and metabolites such as bradykinin, prostaglandins, and hydrogen ions. During blood flow restriction, CO_2_ accumulates due to cellular metabolism. CO_2_ and its associated hydrogen ions may reduce the intracellular pH and inhibit calcium channel activity, resulting in smooth muscle relaxation [16-19]. As shown in previous studies [1,2], a cuff-induced squeeze-hold can relax precapillary sphincters, activate dormant capillary networks, and improve circulation. This helps to flush pain-related substances and raise pain thresholds, thereby providing relief. Unlike massage or stretching, squeeze-hold therapy effectively targets the microvascular level and offers a potentially superior method for reducing DOMS. Second, the mechanical compression by the pneumatic cuff likely activated large-diameter afferent fibers (Aβ fibers), which inhibited nociceptive transmission from Aδ and C fibers at the spinal dorsal horn according to the gate control theory, thereby producing immediate analgesia. This neurophysiological pathway, by inhibiting the transmission of nociceptive signals at the spinal level [20-22], may contribute to the immediate relief of soreness observed in this study. However, both mechanisms are inherently short-lived. Once the mechanical stimulus was removed, the gate control effect disappeared, and newly produced inflammatory cytokines continued to accumulate.

Previous studies have shown that the inflammatory response following eccentric exercise peaks between 24 and 48 hours, during which IL-1β, IL-6, and TNF-α remain elevated [10,23,24]. Moreover, CRP, a systemic marker of inflammation, has been reported to stay elevated for 24-48 hours post-exercise before gradually returning to baseline [25,26]. These findings support the interpretation that the recurrence of soreness after 48 hours was due to persistent inflammatory mediator production, despite the temporary clearance achieved by squeeze-hold.

**Figure 6:**
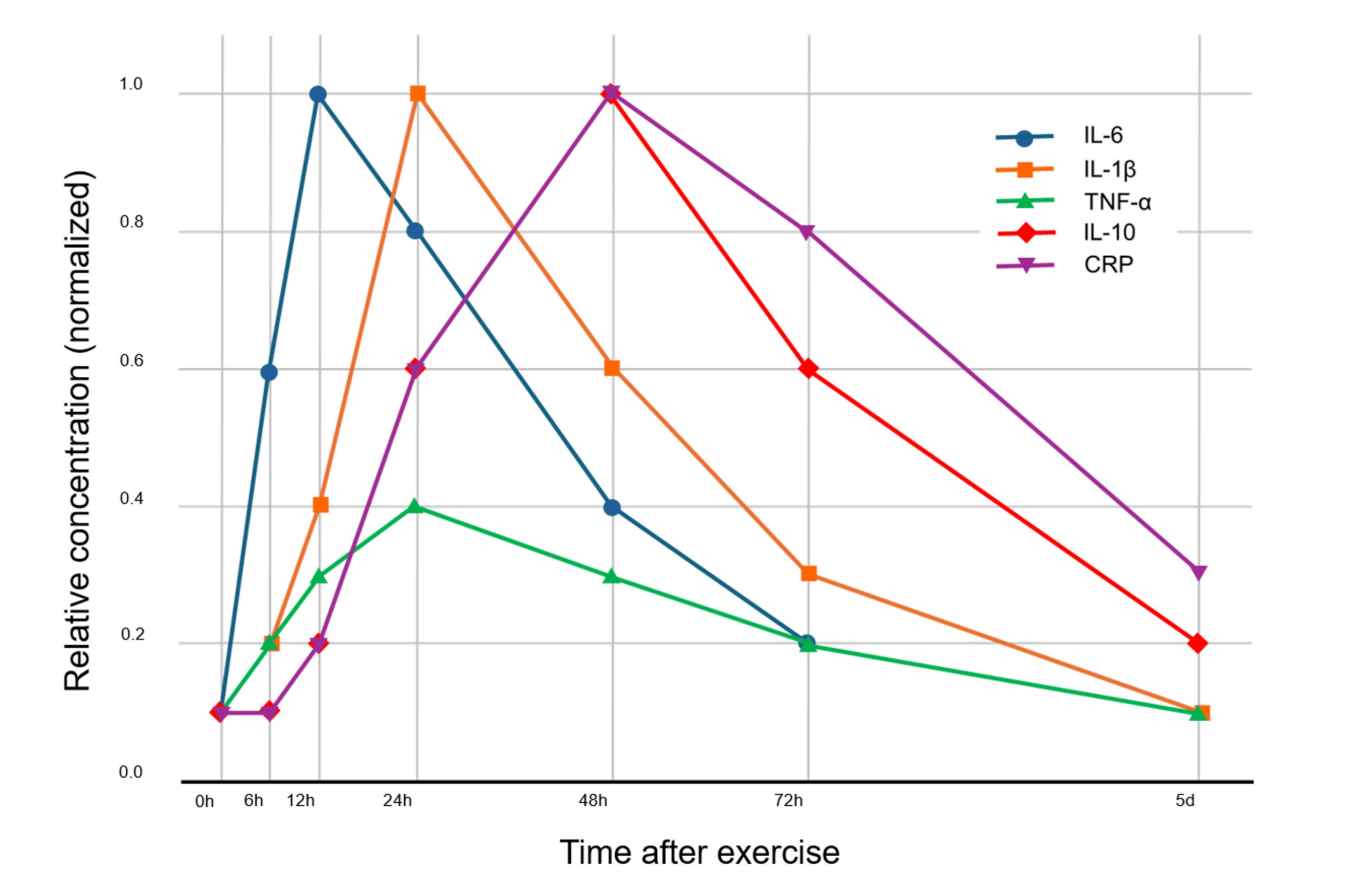
Time course of inflammatory markers (IL-6, IL-1β, TNF-α, IL-10, and CRP) after DOMS-inducing exercise IL-6 rises rapidly and peaks within hours before declining by 24-48 hours; IL-1β peaks around 24 hours and decreases by 48-72 hours; TNF-α shows only a mild transient increase; IL-10 rises 24-48 hours as an anti-inflammatory cytokine; CRP peaks at 24-48 hours and returns to baseline within 3-5 days [23-26]. DOMS: delayed-onset muscle soreness

Therefore, it may be hypothesized that repeated application of the squeeze-hold technique during the period of sustained systemic inflammation, as reflected by elevated CRP levels 24-48 hours post-exercise, could enhance the clearance of inflammatory mediators and provide more consistent pain relief compared to a single intervention. To aid interpretation, I created a schematic figure (Figure 6) based on previous studies describing the time course of inflammatory markers following eccentric exercise [23-26]. This figure illustrates the approximate peak timing and duration of changes in IL-6, IL-1β, TNF-α, IL-10, and CRP, highlighting that the inflammatory response persists for 24-48 hours, which corresponds to the period when our intervention was applied.

Compared to other commonly used modalities such as cryotherapy or transcutaneous electrical nerve stimulation (TENS), squeeze-hold therapy offers the unique advantage of improving microcirculatory dynamics in both superficial and deeper muscular layers. While cryotherapy primarily acts through vasoconstriction and reduction of nerve conduction velocity [27-29], it does not actively enhance perfusion after the intervention. In contrast, the reperfusion effect following squeeze-hold compression may provide both metabolic clearance and improved oxygenation to the affected area. 

Perspectives and significance

This study focused on the immediate effects of the squeeze-hold therapy. Future research should explore its time-dependent impact and effectiveness in the management of chronic muscle soreness. The effective alleviation of DOMS may improve training efficiency and recovery in sports settings. In particular, investigating the optimal pressure, duration, and frequency of intervention may provide important insights for tailoring protocols in both athletic and rehabilitation settings. Moreover, identifying responder characteristics (e.g., baseline vascular reactivity or pain sensitivity) could help to personalize the treatment. From a clinical perspective, the simplicity and non-invasiveness of squeeze-hold therapy make it an attractive option for early-phase rehabilitation or return-to-play protocols. It could be particularly useful in populations where active recovery is limited due to pain or fatigue. In the future, integration of portable pneumatic devices could allow self-administered interventions, expanding its accessibility beyond clinical environments.

Limitations of the study

This study did not compare squeeze-hold therapy with other common treatments, such as massage or stretching. However, further studies with larger sample sizes and longitudinal designs are required to confirm these findings.

## Conclusions

This preliminary study demonstrated that a single session of squeeze-hold therapy using a pneumatic cuff significantly reduced DOMS in healthy individuals. The intervention produced a short-term effect, suggesting its potential utility as a rapid, non-invasive strategy for pain relief following eccentric exercise. The underlying mechanisms likely involve improved microcirculation and modulation of nociceptive signaling through both vascular and neurophysiological pathways. However, the effect disappeared after another 24 hours. The recurrence of pain may be due to the sustained production of inflammatory mediators. These findings suggest that repeated applications during the 24-48-hour period of elevated CRP may provide more consistent analgesic benefits. Given its simplicity and portability, squeeze-hold therapy may be a valuable addition to recovery protocols in both clinical rehabilitation and athletic settings. Further studies with larger sample sizes, longer follow-up periods, and comparisons to other standard interventions are warranted to validate and optimize its clinical application.
